# Effect of culture conditions on the performance of lignocellulose-degrading synthetic microbial consortia

**DOI:** 10.1007/s00253-021-11591-6

**Published:** 2021-10-01

**Authors:** Yanfang Wang, Theo Elzenga, Jan Dirk van Elsas

**Affiliations:** 1 Cluster of Microbial Ecology, Groningen Institute for Evolutionary Life Sciences, University of Groningen, Groningen, the Netherlands; 2 Plant Physiology Group, Groningen Institute for Evolutionary Life Sciences, University of Groningen, Groningen, the Netherlands

**Keywords:** Lignocellulose, Degradation, Synergism, Bacterial–fungal consortia

## Abstract

In this study, we examined a synthetic microbial consortium, composed of two selected bacteria, i.e., *Citrobacter freundii* so4 and *Sphingobacterium multivorum* w15, next to the fungus *Coniochaeta* sp. 2T2.1, with respect to their fate and roles in the degradation of wheat straw (WS). A special focus was placed on the effects of pH (7.2, 6.2, or 5.2), temperature (25 versus 28 °C), and shaking speed (60 versus 180 rpm). *Coniochaeta* sp. 2T2.1 consistently had a key role in the degradation process, with the two bacteria having additional roles. Whereas temperature exerted only minor effects on the degradation, pH and shaking speed were key determinants of both organismal growth and WS degradation levels. In detail, the three-partner degrader consortium showed significantly higher WS degradation values at pH 6.2 and 5.2 than at pH 7.2. Moreover, the two bacteria revealed up to tenfold enhanced final cell densities (ranging from log8.0 to log9.0 colony forming unit (CFU)/mL) in the presence of *Coniochaeta* sp. 2T2.1 than when growing alone or in a bacterial bi-culture, regardless of pH range or shaking speed. Conversely, at 180 rpm, fungal growth was clearly suppressed by the presence of the bacteria at pH 5.2 and pH 6.2, but not at pH 7.2. In contrast, at 60 rpm, the presence of the bacteria fostered fungal growth. In these latter cultures, oxygen levels were significantly lowered as compared to the maximal levels found at 180 rpm (about 5.67 mg/L, ~ 62% of saturation). Conspicuous effects on biomass appearance pointed to a fungal biofilm–modulating role of the bacteria.

**Key points**

• *Coniochaeta sp. 2T2.1 has a key role in wheat straw (WS) degradation.*

• *Bacterial impact shifts when conditions change.*

• *pH and shaking speed are key drivers of the growth dynamics and WS degradation.*

**Supplementary Information:**

The online version contains supplementary material available at 10.1007/s00253-021-11591-6.

## Introduction

There is an increasing demand of renewable substitutes for liquid fuels as well as building blocks for industry, and this has promoted the use of lignocellulosic biomass (LCB) as a source of carbonaceous compounds (Zhang et al. 2014; Jiménez et al. 2015). LCB mainly consists of three types of biopolymers: cellulose (35–50 wt. %), hemicellulose (20–35 wt. %), and lignin (5–30 wt. %), next to varied amounts of starch and pectin (Zhang and Lynd 2004). This implies that nearly 75% polysaccharide sugar is contained in many LCB sources (Van Dyk and Pletschke 2012). Such sources include agricultural waste products, e.g., wheat straw (WS), corn stover, sugarcane bagasse, and cut wood, next to municipal waste. Moreover, dedicated energy crops, such as miscanthus and switchgrass, are widely used as “green” sources of LCB (Gomez et al. 2008). In attempts to make use of LCB, its conversion into monomers often constitutes a prime bottleneck, hampering applications. Hence, most processes nowadays have a physicochemical (pretreatment) stage, next to a biological (bioconversion) stage. However, current physicochemical pretreatment methodologies (alkaline, acid, and thermal treatments) for improving LCB degradation not only increase the cost, but also hamper downstream processes. Great interest has therefore been placed in the further development of biological treatment processes based on lignocellulolytic microorganisms and/or their enzymes (Jiménez et al. 2014; Cortes-Tolalpa et al. 2017; Maruthamuthu et al. 2016).

For LCB bioconversion, diverse enzymes, i.e., lytic polysaccharide monooxygenases (LPMOs), laccases, xylanases, arabinofuranosidases, cellobiohydrolases, endoglucanases, and β-glucosidases, are required. Therefore, the biodegradation rates achievable with single strains are often reported to be unsatisfactory, as crucial parts of the required enzymatic machinery may be absent. LCB degradation by microbial consortia that have a greater enzymatic palette and dynamic expression range, has received more attention recently (Cortes-Tolalpa et al. 2016; Jiménez et al. 2014), with particular combinations of bacteria and fungi offering great potential. The finding that such microbial consortia outperform single organisms is consistent with the contention that, in nature, degradation processes appear like microbial “group efforts.” In LCB-degrading consortia, the different organisms may even develop symbiotic or synergistic relationships with each other during the lignocellulose degradation process (Cragg et al. 2015; Jiménez et al. 2017; Cortes-Tolalpa et al. 2017). One obvious interaction is exemplified by the fact that the enzymes secreted by primary cellulose degraders break the cellulose down into monomers or oligomers like cellodextrins, cellobiose, and glucose. These compounds may be further transformed and assimilated by other — saccharolytic — microbes, thus removing feedback inhibition processes. Hence, primary (hemi)cellulose degradation by one organismal type may be followed by removal of the breakdown products by other organisms (Cortes-Tolalpa et al. 2020). Another key interaction is that between so-called synergistic microbes, which produce complementary enzymes that work jointly in order to better open up and thus degrade the substrate (Jiménez et al. 2017). The latter effect has been coined “Division of Labor” and is a promising area of research.

In previous work performed in our lab, microbial consortia derived from naturally occurring microbial communities, encompassing specific biodegradative bacteria (mainly affiliated to the enteric bacteria *Citrobacter* and *Klebsiella*, as well as *Sphingobacterium*, *Flavobacterium*, and *Acinetobacter*) and fungi (such as *Coniochaeta* and *Trichosporon*), were selected as key LCB degraders (Jiménez et al. 2015; Cortes-Tolalpa et al. 2016). In these studies, inoculum source and type of substrate proved to be the key determinants of the composition of the microbial degrader consortia, yet with varying enzyme activities. Different LCB-degrading consortia have thus become available, as derived from various initial microbiomes from diverse substrates. Remarkably, although the composition of the microbial consortia varied, some consistently occurring “core” microorganisms were discerned. Further work revealed that some of these core organisms showed synergism when growing on WS, as compared to glucose (Cortes-Tolalpa et al. 2017). Very interestingly, in shaken cultures at pH 7.2, consortia composed of two bacteria (*Citrobacter freundii* so4 and *Sphingobacterium multivorum* w15) and one fungus (*Coniochaeta ligniaria*) showed superior performance and synergism (Cortes-Tolalpa et al. 2017). Although additional recent work in our laboratories (Jimenez et al. 2020) provided preliminary data pointing at a major role for the fungal partner, it remains unknown to what extent the synergism is influenced by the (abiotic) conditions in the cultures and whether such constructed simple consortia can explain the performance of original multispecies consortia. Clearly, a better understanding of the dynamics and performance (synergism) within the consortia and how these are affected by the conditions established in the culture is of great value for further applied research and development.

Here, we hypothesize that (1) constructed simplified microbial consortia can explain, to a major extent, the functioning of the complex WS-degrading microbial consortia, and (2) abiotic conditions, in particular pH and shaking speed (affecting oxygen level and distribution), determine the ecological opportunities of the consortium members and hence the outcome (efficiency) of the degradative process. The study was built around a novel consortium composed of the recently sequenced bacteria *C. freundii* so4 and *S. multivorum* w15 (Cortes-Tolalpa et al. 2020) and the fungus *Coniochaeta* sp. 2T2.1 (Mondo et al. 2019).

## Materials and methods

### Strains, microbial consortia, and growth conditions

#### Strains and consortia

*C. freundii* so4 and *S. multivorum* w15 have previously been described as members of microbial consortia able to degrade raw wheat straw (WS; Cortes-Tolalpa et al. 2016). Both strains were able to grow on raw WS as the sole carbon source (Cortes-Tolalpa et al. 2017), in cultures here denoted as “S” and “W”. In addition, *Coniochaeta* sp. 2T2.1, originally isolated on potato dextrose agar (PDA, Sigma-Aldrich, Darmstadt, Germany) from a lignocellulolytic microbial consortium (Jiménez et al. 2014), was grown on WS, in cultures denoted “T”. The here-used synthetic consortia were composed as follows: SWT — *C. freundii* so4, *S. multivorum* w15, and *Coniochaeta* sp. 2T2.1, and SW — *C. freundii* so4/*S. multivorum* w15. Moreover, the original LCB-degrader consortium (coined “T10”) was produced from forest soil via a dilution-to-stimulation approach, on WS as the substrate (Cortes-Tolalpa et al. 2016). Here, it was recovered from − 80 °C stocks, cultured on WS as the substrate at 28 °C for 10 days, and then used as the inoculum for the experiments. The bacterial strains used in this study have been deposited in the German Collection of Microorganisms and Cell Cultures (DMSZ, Braunschweig, Germany). *C. freundii* so4 is deposited under the number DSM 106340 T; strain w15, identified as a member of the species *S. multivorum*, is deposited under the number DSM 106342. We are in the process of re-identifying strain w15 as a member of the new species *S. paramultivorum* (Wang et al. in review), but for the purpose of this study, we prefer to keep the original designation, i.e., *S. multivorum*. The fungal strain 2T2.1, identified as a *Coniochaeta* sp., has been deposited at the United States Department of Agriculture (USDA) Agricultural Research Service (ARS) Northern Regional Research Laboratory Open Culture Collection (NRRL, Peoria, IL, USA), under the accession number NRRL Y-64006.

#### pH tolerance ranges

The pH tolerance ranges of growth of each strain were determined as in Supplementary Material. *C. freundii* so4 grew in the pH range 5.0–10.0, was strongly inhibited at pH 4.0, and did not survive at pH 3.0 (Supplemental Fig. S1a). *S. multivorum* w15 grew at pH 5.0–7.0 (optimally at pH 7.0) and did not survive at pH values outside of this range (using one pH unit steps) (Supplemental Fig. S1b). *Coniochaeta* sp. 2T2.1 grew well in the pH range 5.2–7.2.

### WS preparation

Fresh WS was obtained, as one batch, from a local farm (Groningen, the Netherlands). It was air-dried in an oven (50 °C) before cutting it into pieces of about 5-cm length. Then, the pieces were thoroughly ground, using a mill hammer, to pieces ≤ 1 mm in order to increase the surface to volume ratio. A thorough wash of this WS, as detailed hereunder, was applied to maximize the removal of water-soluble organic compounds. For all experiments, the ground WS was washed by treating each 50 g twice with 1.5 L of distilled water, followed by filtering over a 210-µm mesh filter. Following this, the substrate was dried at 50 °C for 48 h and kept for further experiments. To assess the effect of the washing treatment, we compared the dynamics of microbial growth (SW and SWT consortia) in mineral media supplemented with washed versus unwashed WS. After 24-h cultivation, the bacterial cell densities in both consortia developing on washed WS increased from 6.5 to ~ 8.5 log cells/mL. In contrast, those in consortia on unwashed WS reached significantly (*p* < 0.05, *T* test) higher levels, from initially 6.3 to up to 9.1 log cells/mL (Supplemental Fig. S2). With respect to fungal growth (SWT consortium), such a difference was not found.

#### Preparation of WS-supplemented culturing flasks

Aliquots of pretreated WS (1%, w/v) were placed in 25 mL mineral medium in Erlenmeyer flasks, which were autoclaved at 121 °C for 27 min before use. The mineral medium contained 7 g/L Na_2_HPO_4_•2H_2_O; 2 g/L K_2_HPO_4_; 1 g/L (NH_4_)_2_SO_4_; 0.1 g/L Ca(NO_3_)_2_•4H_2_O; and 0.2 g/L MgCl_2_•6H_2_O g/L, and was set at pH 7.2, 6.2 or 5.2 (Jiménez et al. 2014; de Lima Brossi et al. 2016; Cortes-Tolalpa et al. 2016). It was supplemented with 25 μL vitamin solution (0.1 g Ca-pantothenate, 0.1 g cyanocobalamin, 0.1 g nicotinic acid, 0.1 g pyridoxal, 0.1 g riboflavin, 0.1 g thiamin, 0.01 g biotin, 0.1 g folic acid; H_2_O 1 L) and 25 μL trace metal solution (2.5 g/L EDTA; 1.5 g/L FeSO_4_•7H_2_O; 0.025 g/L CoCl_2_; 0.025 g/L ZnSO_4_•7H_2_O; 0.015 g/L MnCl_2_; 0.015 g/L NaMoO_4_•2H_2_O; 0.01 g/L NiCl_2_; 0.02 g/L H_3_BO_3_; 0.005 g/L CuCl_2_). Sterility of the medium was verified by plating an aliquot on trypticase soy agar (TSA) plates, and scoring plates for colony growth after appropriate incubation. All chemicals and reagents used in this study were of analytical molecular biology grade (Sigma-Aldrich, Darmstadt, Germany).

### Estimation of WS degradation by weight loss measurements

At the end of each culture, residual solid WS matter was retrieved, washed twice with distilled water, and filtered through filter paper (Cat. no. 516–0304, VWR International Europe BV, Amsterdam, The Netherlands). It was then dried at 50 °C for 48 h. Following drying, the weight of the residual matter was measured and compared to that in a control treatment (without inoculum). In the control, 90–95% of the initial weight was recoverable and so data were corrected for this loss. The percentage weight loss was defined as the ratio of the dry weight loss compared to the initial dry weight (%) as given by the following formula:

$$\mathrm{Substrate}\;\mathrm{weight}\;\mathrm{loss}\;\left(\%\right)=\:\left[\left(a\:-\:b\right)/c\right]\:\times\:100$$

where *a* is the residual control substrate weight, *b* is the residual substrate weight, and *c* is the total initial substrate weight (de Lima Brossi et al. 2016). Statistical comparisons of the samples’ substrate weight losses were performed using one-way ANOVA of the means per treatment (Tukey’s test) using SPSS (IBM, Armonk, NY). RStudio (version 1.4.1106, RStudio Team 2021) software was used to create the weight loss figures.

In comparisons of weight losses across different conditions (pH, temperature, shaking speed), the % of maximal (max) weight loss was used, as calculated by the following formula:

$$\%\;\mathrm{of}\;\max\;\mathrm{weight}\;\mathrm{loss}=\left(\mathrm{substrate}\;\mathrm{weight}\;\mathrm{loss}/\max\;\mathrm{weight}\;\mathrm{loss}\right)\:\times\:100$$

where the highest weight loss achieved within the comparison was used as the 100% max weight loss value. Thus, the max weight loss values in each experiment varied and they were mentioned in the “11” section.

### Microbial cultures and growth measurements

Cultures were grown in 100-mL Erlenmeyer flasks containing 25 mL media (in triplicate per treatment). To prepare the inocula, bacterial strains were recovered from − 80 °C stocks on TSA plates at 28 °C for 48 h, whereas the fungal strain was pre-grown on PDA plates at 28 °C for 72 h. Then, to produce starter cultures for the experiments, fresh colonies of each strain were transferred into LB (Sigma-Aldrich, Darmstadt, Germany) and potato dextrose (PD, Sigma-Aldrich, Darmstadt, Germany) media at 28 °C for 18 h (bacteria) or 48 h (fungus). The cell densities of the bacteria and fungus (separate propagules) were then estimated in the resulting starter cultures, by microscopy using a Bürke-Turk chamber (Blaubrand®, Wertheim, Germany) according to a standard protocol. Following this, the starter cultures were diluted and cells were added to the respective media at about 6 log cells per mL.

The experiments were performed in three phases, testing, in a comparative fashion, different culture conditions (temperature, pH, and shaking speed). The temperatures used were 25 versus 28 °C; the pH values were 7.2, 6.2, or 5.2; and the shaking speeds were 180 versus 60 rpm. The shaker/incubator used was the Multitron Pro and the Ecotron (both from INFORS HT, Bottmingen, Switzerland); the rotary motion of two shakers was comparable. Microbial growth was measured at regular time intervals (as shown in the figures), until the end of each of the experiments. At each time point, 1 mL was harvested from the cultures and used for either direct cell counting by Bürke-Turk chamber measurements, or for (serial) dilution plating on TSA plates supplemented with 0.05 mg/mL cycloheximide for enumerating the colony forming units (CFUs) of each bacterial strain (easily discerned by different colony morphologies) and on TSA plates containing 0.05 mg/mL streptomycin, 0.05 mg/mL chloramphenicol, and 0.05 mg/mL tetracycline) for enumerating fungal colonies. The inoculated plates were incubated at 28 °C for, at least, 24–72 h, after which the developed colonies were counted. To determine the growth rates of the cultures (μ, h^−1^), the numbers of CFUs measured were log-transformed and the slope of each growth curve was determined.

### Measurement of dissolved oxygen

After 24 and 72 h of incubation, 1-mL samples were collected from the culture liquids (avoiding WS particles), and immediately used to measure the oxygen levels using a liquid-phase oxygen electrode. The oxygen levels were detected polarographically by an S1 Clark-Type electrode (González et al. 2001).

## Results

### WS degradation and population dynamics in constructed versus complex degrader consortia at neutral pH

#### WS degradation

**Fig. 1:**
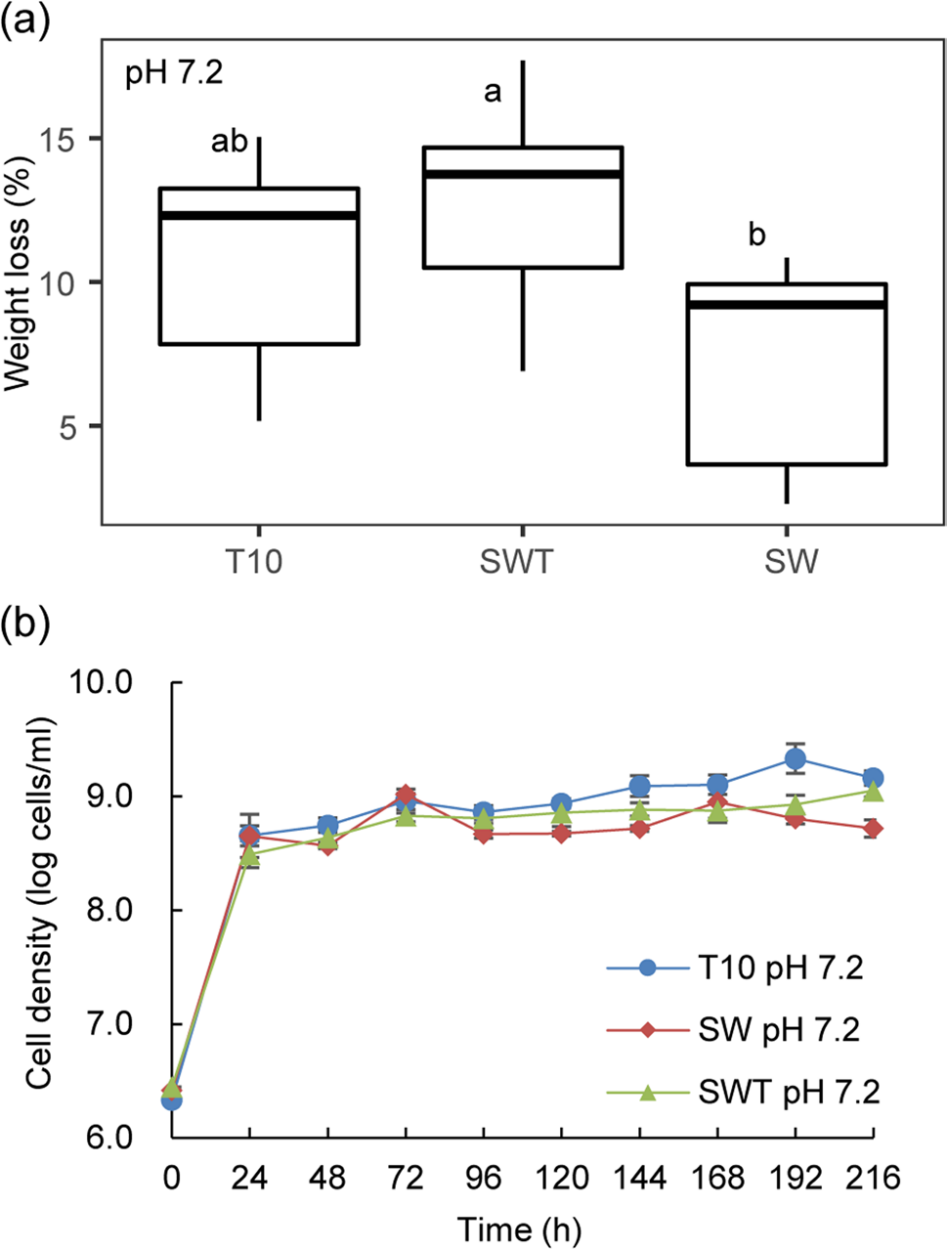
Comparison of wheat straw degradation (a) and growth dynamics (b) between synthetic consortium SWT and complex consortium T10 at (original) pH 7.2. Abbreviations: T10, forest soil-derived LCB-degrader consortium (10 transfers; Cortes-Tolalpa et al. 2016); SW, consortium consisting of *Citrobacter freundii* so4 and *Sphingobacterium multivorum* w15; SWT, consortium of strains so4, w15 and *Coniochaeta* sp. 2T2.1

In this initial experiment, we examined to what extent the selected simplified degrader consortia SW (*C. freundii* so4/*S. multivorum* w15) and SWT (*C. freundii* so4/*S. multivorum* w15/C*oniochaeta* sp. 2T2.1) could explain the performance of the original soil-derived LCB-degrader consortium T10 (containing 228 ± 12 bacterial OTUs and an estimated tens of fungal types; Cortes-Tolalpa et al. 2016), at the original pH (7.2). Remarkably, the SWT consortium revealed WS degradation performance similar to that of the T10 one, with 12.82 ± 1.93% of wheat straw being consumed after 10 days (*p* > 0.05, Fig. 1a). In contrast, the WS degradation performance of the SW consortium was inferior (Fig. 1a), with only 7.49 ± 0.68% WS degradation being recorded after 10 days.

#### Population dynamics

In all aforementioned cultures, rapid microbial growth on WS was detected within the first 24 h of cultivation. Irrespective of the system, the bacterial cell densities increased from, initially, around 4 × 10^6^ to about 5 × 10^8^ cells/mL. The initial period of rapid growth was followed by a period of slow population size increases (up to 10 days), with stabilization of the total bacterial cell numbers at about 1–2 × 10^9^ cells/mL in the SWT and T10 consortia, and at about 7 × 10^8^ cells/mL in the SW ones (Fig. 1b). The fungal cell densities were roughly estimated to be in the 10^5^ (propagules/mL) range. Overall, although differences in the final cell densities were found between, on one hand, the SWT/T10 (SWT = T10, *p* > 0.05; SWT: 1 × 10^9^ cells/mL; T10: 2 × 10^9^ cells/mL) and, on the other hand, the SW consortia (SWT = T10 > SW, *p* < 0.01; SW: 7 × 10^8^ cells/mL), these were not large.

### Effect of temperature on WS degradation and population dynamics in synthetic versus complex consortia

In a second experiment, we examined whether temperature (25 versus 28 °C) would affect the behavior and performance of the constructed versus the complex LCB-degrader consortia (Cortes-Tolalpa et al. 2016), over 10 days. The two temperatures previously used to select degradative consortia on lignocellulose (25 and 28 °C) were tested (Cortes-Tolalpa et al. 2016; Jiménez et al. 2015). The T10 consortium performed better at 25 °C (10.30 ± 2.95% WS weight loss/10 days) than at 28 °C (6.45 ± 1.35%), whereas consortia SW and SWT achieved higher performance at 28 °C, with 4.78 ± 2.78% and 8.32 ± 0.03% WS weight loss respectively, compared to 1.98 ± 0.37% and 5.32 ± 1.96% at 25 °C (Supplemental Fig. S3). Very fast microbial growth on WS was detected within the first 24 h of cultivation, irrespective of the system or temperature, and population sizes of 4 × 10^9^ cells/mL at 28 °C, and 1 × 10^9^ cells/mL at 25 °C were reached (Supplemental Fig. S4). Thus, within this narrow range, temperature had a contrasting effect on the constructed and complex degrader consortia, with 28 °C supporting higher population sizes. In all further experiments, we used 28 °C as the standard temperature.

### Effect of pH on WS degradation in synthetic versus complex consortia

In a next experiment, we examined whether reduction of pH (using pH 6.2 and pH 5.2, next to the control pH 7.2) would affect the relative behavior and performance of the constructed versus the complex (T10) LCB-degrader consortia, over 10 days.

#### Effect of pH on WS degradation

**Fig. 2:**
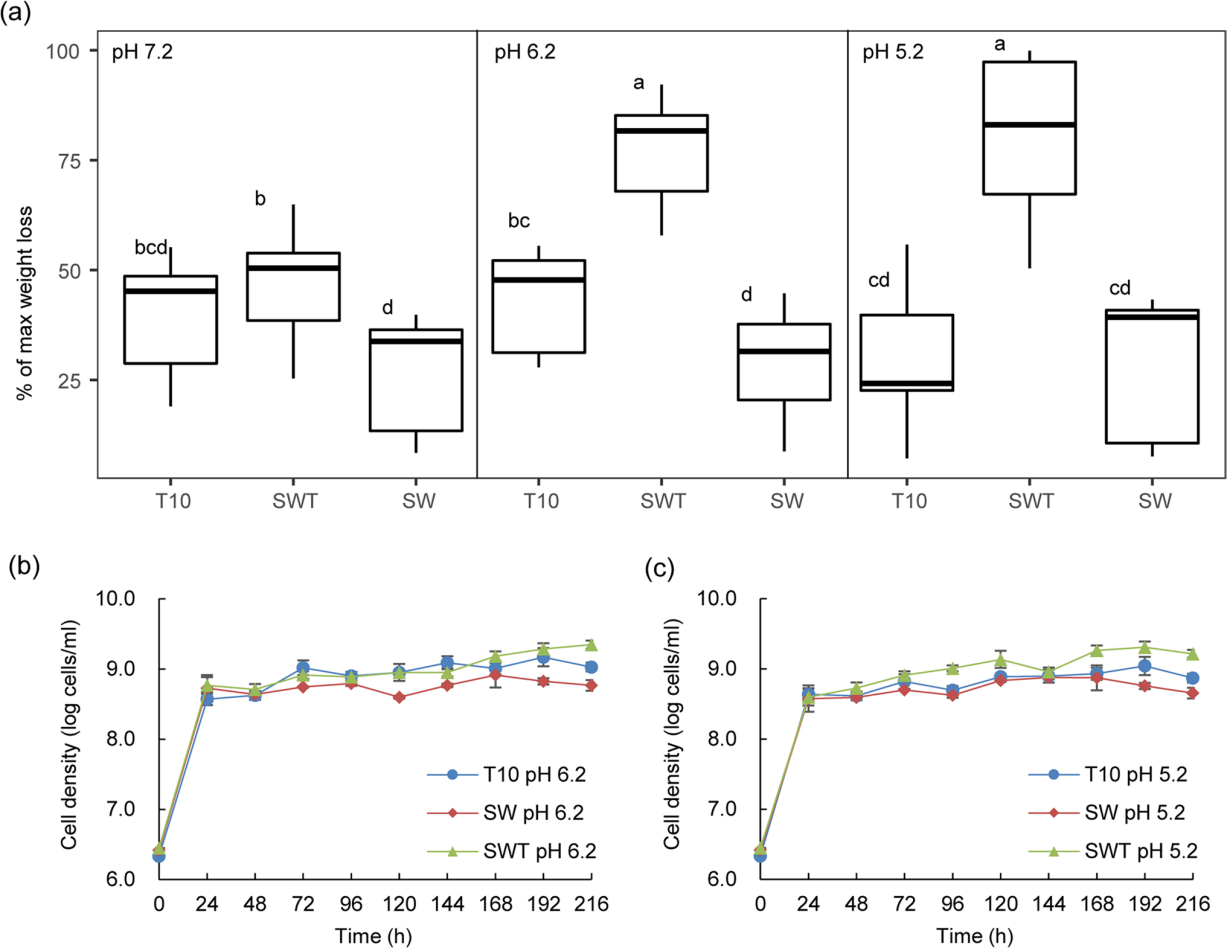
Effect of pH on wheat straw degradation (a) and growth dynamics (b and c) in synthetic consortium SWT versus complex consortium T10. Abbreviations: T10, forest soil-derived LCB-degrader consortium (10 transfers; Cortes-Tolalpa et al. 2016); SW, consortium consisting of *Citrobacter freundii* so4 and *Sphingobacterium multivorum* w15; SWT, consortium of strains so4, w15 and *Coniochaeta* sp. 2T2.1

In contrast to the similar WS degradation values between SWT and T10 at pH 7.2 (*p* > 0.05), the SWT consortia significantly outperformed the T10 ones at both pH 6.2 and 5.2 (*p* < 0.001). (See Fig. 2a.) In detail, the SWT degradation values amounted to up to 22.38 ± 2.48% at pH 5.2 (21.09 ± 1.38% at pH 6.2), being only 12.81 ± 1.93% at pH 7.2. In contrast, only small amounts of the WS were consumed by the SW consortium (7.82 ± 2.80% at all three pH values). Consistent with our previous findings (Jimenez et al. 2020), this suggests that C*oniochaeta* sp. 2T2.1 has a major role in the WS degradation performance, being fortified at lower pH values; the role of the bacteria might be described as accessory (Fig. 2a).

#### Effect of pH on population dynamics

The growth dynamics at lowered pH (pH 6.2 and pH 5.2) were largely similar to those at pH 7.2, with slow population size increases following an initial rapid growth period. At both pH values, the total bacterial cell numbers stabilized at about 2–4 × 10^9^ cells/mL in the SWT cultures, at about 9–10 × 10^8^ cells/mL in the T10 ones, and at 7–8 × 10^8^ cells/mL in the SW ones (Fig. 2b). Thus, significant differences in the final cell densities were found between the SWT, T10, and SW consortia at both pH 6.2 (SWT: 4 × 10^9^ cells/mL; T10: 1 × 10^9^ cells/mL; SW: 8 × 10^8^ cells/mL) and pH 5.2 (SWT: 2 × 10^9^ cells/mL; T10: 9 × 10^8^ cells/mL; SW: 7 × 10^8^ cells/mL) (SWT > T10 > SW, *p* < 0.05) (Fig. 2b).

### WS degradation and population dynamics in synthetic tri- (SWT), bi- (SW), and monoculture (S, W, T) degrader cultures — effect of pH

To unravel the role of each organism in the synthetic consortia or monocultures, we examined the degradation performance and population dynamics in the SWT, SW, S, W, and T cultures, at three pH values (shaken at 180 rpm, 28 °C).

#### pH shifts in the different treatments

All mono- and bi-bacterial culture setups at initial pH values of 7.2, 6.2, and 5.2 revealed stable pH for over 10 days. In contrast, all cultures containing *Coniochaeta* sp. 2T2.1 revealed pH downshifts. The pH values of the pH-7.2 and pH-6.2 cultures dropped to, respectively, 6.9 and 5.8 (after 10 days). Stronger downshifts were recorded for the pH-5.2 cultures containing strain 2T2.1 (to ~ pH 4.0; Supplemental Fig. S5).

#### WS degradation in the different treatments

**Fig. 3:**
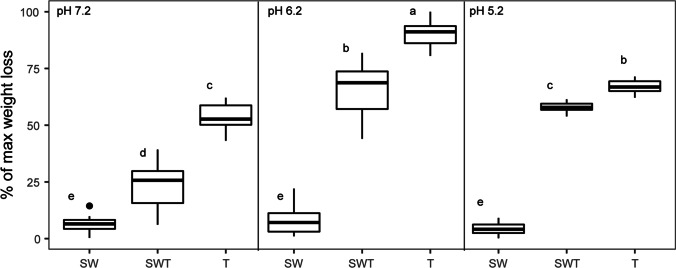
Effect of pH on wheat straw degradation in synthetic consortia. Abbreviations: SW, consortium consisting of *Citrobacter freundii* so4 and *Sphingobacterium multivorum* w15; T, monoculture of *Coniochaeta* sp. 2T2.1; SWT, consortium of strains so4, w15 and 2T2.1

At all pH values, the SWT consortia showed significantly higher WS degradation values (*p* < 0.05) than the SW biculture and the S and W monocultures (without fungal strain 2T2.1). In fact, the degradation values in the latter ones remained low and were relatively unaffected by pH. In detail, at all tested pH values, the T (control strain 2T2.1) cultures showed the highest WS degradation values compared to SWT, SW, and both bacterial monocultures (13.84 ± 1.53% at pH 7.2, 23.27 ± 1.66% at pH 6.2, and 17.24 ± 0.74% at pH 5.2). Similar to this, WS degradation was significantly different (*p* < 0.05) within the SWT treatments across the three pHs, being highest at pH 6.2 (16.68 ± 3.27%), followed by pH 5.2 (14.83 ± 0.61%) and finally pH 7.2 (6.10 ± 2.85%) (Fig. 3). In contrast, the bacterial bi- (Fig. 3) and monocultures (data not shown) revealed low degradation performance and no clear effect of pH.

#### Population dynamics in the different treatments

##### pH 7.2

**Fig. 4:**
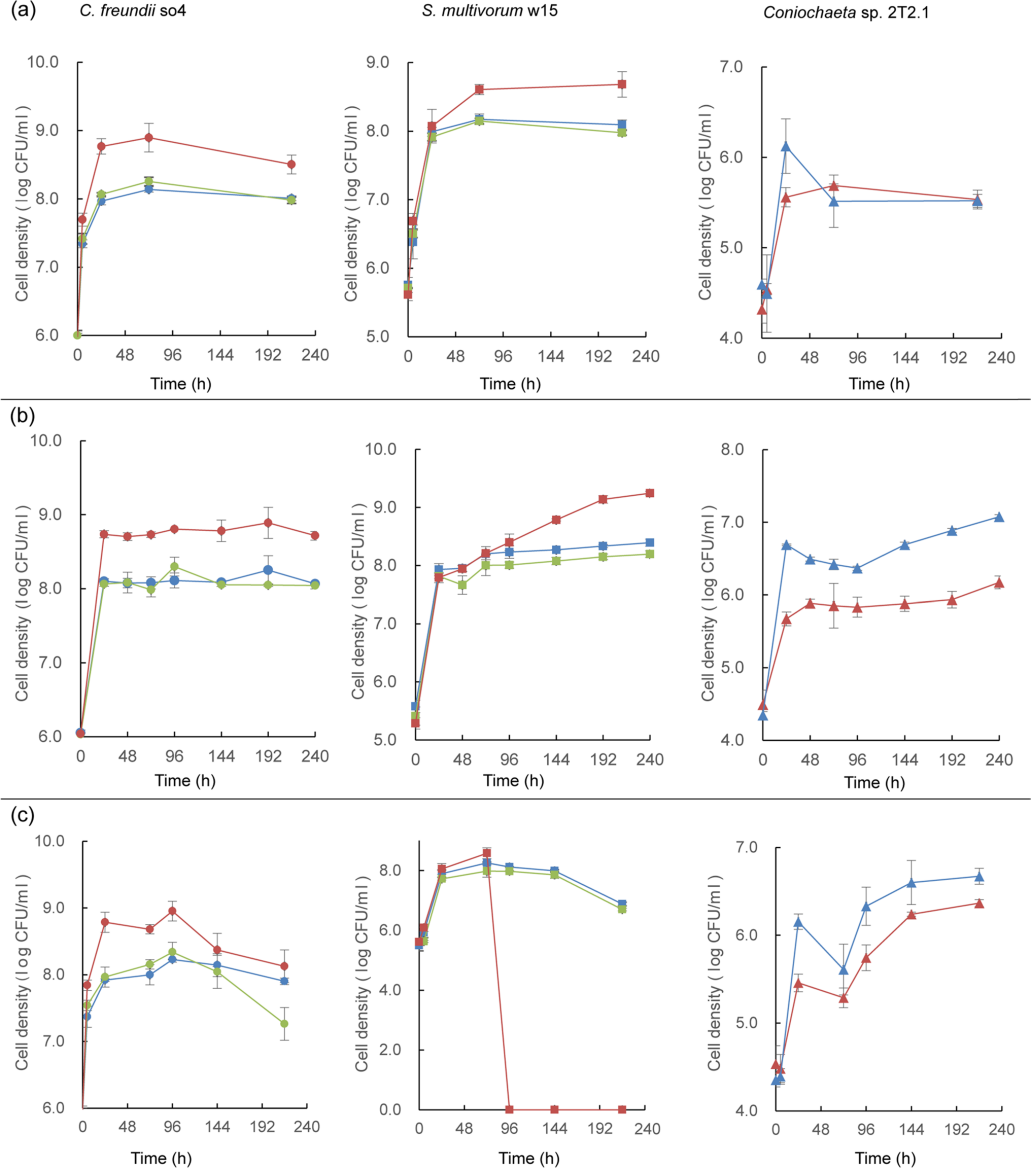
Effect of pH on growth dynamics in synthetic consortia. (a) pH 7.2; (b) pH 6.2; (c) pH 5.2. Left: *Citrobacter freundii* so4 (circle); middle: *Sphingobacterium multivorum* w15 (square); right: *Coniochaeta* sp. 2T2.1 (triangle). Blue: strain growing in monoculture; green: strain growing in biculture of *C. freundii* so4 and *S. multivorum* w15; red: strain growing in consortium of strains so4, w15, and 2T2.1

As expected, at pH 7.2, in all bacterial cultures, with or without strain 2T2.1, both bacterial strains showed rapid initial (0–24 h) growth. *C. freundii* so4 grew at rate 0.0995 ± 0.0054 (μ, h^−1^) in the SWT, 0.0767 ± 0.0110 (μ, h^−1^) in the SW, and 0.0688 ± 0.0052 (μ, h^−1^) in the S culture, from about 1 × 10^6^ to about 8 × 10^8^ CFU/mL. Similarly, *S. multivorum* w15 showed μ values of 0.0943 ± 0.0115 (h^−1^) in the SWT, 0.0886 ± 0.0028 in the SW, and 0.0908 ± 0.0044 in the W culture, from about 8 × 10^5^ to about 1 × 10^8^ CFU/mL. These initial growth phases were followed by long periods of slow population size increases (μ slightly over 0.00 (h^−1^)), with stabilization of the cell densities at about 9 × 10^8^ CFU/mL. In contrast, C*oniochaeta* sp. 2T2.1 grew steadily and progressively, initially at μ 0.0522 ± 0.0043 in SWT, and 0.0699 ± 0.0134 in monoculture T. This differential growth rate reverted later, resulting in similar growth from about 5 × 10^4^ to about 5 × 10^5^ CFU/mL (Fig. 4a). Interestingly, in the SWT culture, strain so4 showed an initial growth rate (0.0995 ± 0.0054) that was higher than that of strain w15 (0.0943 ± 0.0115) (0–24 h), which trend inverted after 24 h, with strain w15 growing at μ 0.0056 ± 0.0057 versus so4 at − 0.0016 ± 0.0010. Strains so4 (after 5 h) and w15 (after 72 h) were both significantly (*p* < 0.05) stimulated by strain 2T2.1, as compared to their dynamics in the SW consortia.

##### pH 6.2

At pH 6.2, the two bacterial strains showed growth patterns similar to those observed at pH 7.2, with *C. freundii* so4 having a higher initial growth rate (*p* < 0.05) in the SWT (0.1123 ± 0.0036 (μ, h^−1^)) than in the SW (0.0846 ± 0.0022) consortium (up to 24 h). Following this, the growth rate of so4 in these two consortia remained close to zero. After similar initial growth, *S. multivorum* w15 showed higher growth rates (*p* < 0.05) in the SWT (0.0071 ± 0.0004) than in the SW (0.0021 ± 0.0003) consortia in the period after 24 h. In contrast, the initial (0–24 h) growth of *Coniochaeta* sp. 2T2.1 was significantly (*p* < 0.05) depressed in the SWT culture (0.0492 ± 0.0120, μ, h^−1^), giving 7 × 10^5^ CFU/mL, as compared to that in the T culture (0.0976 ± 0.0025; giving 7 × 10^6^ CFU/mL). This effect remained detectable over 10 days, with growth rates of 0.0016 ± 0.0006 in SWT, and 0.0026 ± 0.0001 in T, and cell density increases to 2 × 10^6^ CFU/mL (SWT) and 1 × 10^7^ CFU/mL (T) (Fig. 4b).

##### pH 5.2

At pH 5.2, *C. freundii* so4 revealed a dichotomic population dynamics akin to that described in the foregoing, with rapid initial followed by extended slow population size increases. In contrast, *S. multivorum* w15 grew well initially but revealed a fast drop of CFU numbers after around 72 h of cultivation. Here, the strain 2T2.1 growth pattern resembled that at pH 6.2, in SWT (0.0420 ± 0.0079 (μ, h^−1^)) showing about half the strain 2T2.1 growth rate in culture T (0.0800 ± 0.0068, μ, h^−1^). Consequently, its cell density was significantly (*p* < 0.05) lowered in the SWT (i.e., 4 × 10^6^ CFU/mL) as compared to the T cultures (about 7 × 10^6^ CFU/mL) at the later stages (Fig. 4c).

Overall, it appeared that *C. freundii* so4 took advantage of the presence of strain 2T2.1 in the SWT culture at all tested pH values, whereas this was true for *S. multivorum* w15 at pH 7.2 and 6.2, but not at pH 5.2. The strain 2T2.1 growth rates were reduced, but not abolished, by the presence of the two bacteria, at all tested pH values, yet to different extents (Fig. 4).

### Effect of shaking speed on WS degradation and population dynamics in the SWT, SW, S, W, and T cultures

In this experiment, we examined the effect of shaking speed (resulting in different prevailing oxygen status, in addition to heterogeneity regarding spatial distributions and dynamics of compounds and cells) across the synthetic consortia at pH 6.2. The gradients of oxygen as well as compounds and cells going from the substrate surface outwards are bound to be different and probably more influential in the low-shaking-speed than those in the high-shaking-speed cultures. Moreover, sites in the culture (surface or bottom) may differ, especially in oxygen. Such heterogeneity may drive all interactions within our consortia. Shaking speed was varied between 180 rpm (high; presumably allowing fast oxygen diffusion into rapidly growing cultures) versus 60 rpm (low; reducing oxygen access and mixing). Oxygen levels were recorded across the treatments.

#### Final pH levels

At the end of the experiment (10 days), the pH values in all cultures run at 60 rpm were lower than those in the corresponding ones run at 180 rpm (Supplemental Fig. S6). In all bacterial mono- and bi-cultures (as well as the negative controls), the values stayed close to the initial ones (i.e., about pH 6.0). In contrast, the SWT and the T cultures revealed final pH values of about 5.5 (at 180 rpm) and 5.26 (at 60 rpm).

#### Oxygen levels

To assess oxygen levels at key points of the growth curves, measurements were made at 24 and 72 h of growth.

After 24 h, the oxygen level in the SW cultures (7.30 mg/L) was similar to that of the negative control (7.51 mg/L) at both 180 rpm and 60 rpm, i.e., slightly below the maximum level of dissolved oxygen in water (i.e., 7.83 mg/L). Clearly, the levels in the SWT cultures (6.75 mg/L) were significantly (*p* < 0.05) lowered. Overall, after 24 h of cultivation, there was no effect of shaking speed on the O_2_ levels in the SWT and SW consortia (Supplemental Fig. S7a). However, the oxygen levels in culture T at 180 rpm (7.44 mg/L) were significantly (*p* < 0.05) higher than in those at 60 rpm (6.29 mg/L).

After 72 h, the SW cultures again showed oxygen levels close to those in the negative controls, at both shaking speeds. At 180 rpm, both the SWT and T cultures showed oxygen levels (7.18 mg/L) slightly below those in the negative controls, indicating the dynamics of O_2_ consumption and diffusion might suffice largely to supply oxygen for microorganisms to grow. However, at 60 rpm, significantly lowered (*p* < 0.05) O_2_ levels were found in these cultures. The SWT culture had the lowest oxygen concentration, i.e., about 5.67 mg/L, followed by the T culture: 6.57 mg/L (Supplemental Fig. S7b).

#### WS degradation

**Fig. 5:**
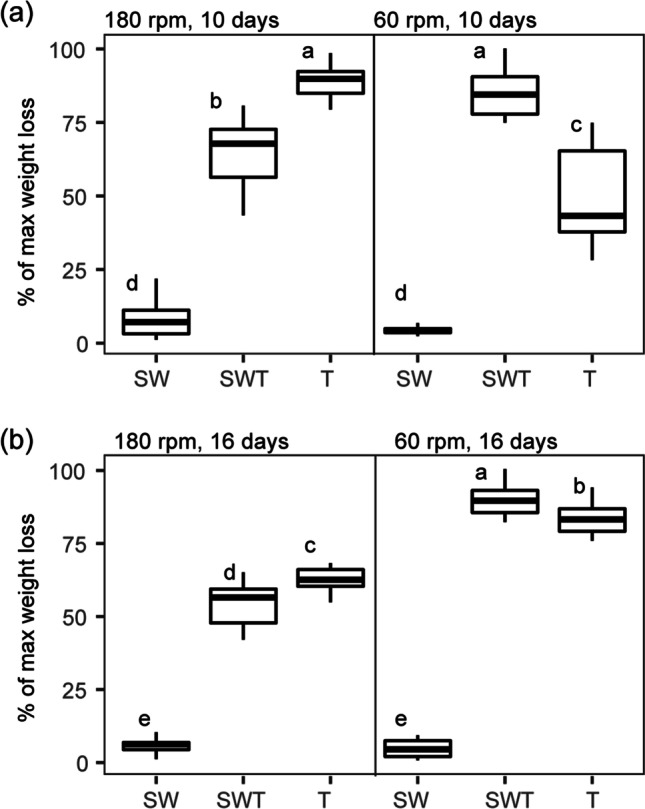
Effect of shaking speed on wheat straw degradation in synthetic consortia. (a) 10 days; (b) 16 days. Abbreviations: SW, consortium consisting of *Citrobacter freundii* so4 and *Sphingobacterium multivorum* w15; T, monoculture of *Coniochaeta* sp. 2T2.1; SWT, consortium of strains so4, w15, and 2T2.1

At both shaking speeds, the SWT cultures, akin to the T ones, showed considerable WS degradation values (180 rpm — 16.68 ± 3.27%; 60 rpm — 22.19 ± 2.19%, Fig. 5a) that even increased to, respectively, 17.94 ± 2.65% and 29.73 ± 1.95%, when the culturing time was extended to 16 days (*p* < 0.05) (Fig. 5b). These degradation values were significantly higher (*p* < 0.05) than those of the SW, S, and W cultures, regardless of culture time (Fig. 5a, b), indicating the importance of the presence of strain 2T21.1. Remarkably, the degradation values at 60 rpm (22.19 ± 2.19%) were significantly (*p* < 0.05) higher than those at 180 rpm (16.68 ± 3.27%) (Fig. 5a). Control culture T achieved 23.27 ± 1.66% WS degradation at 180 rpm, but only 12.70 ± 4.41% at 60 rpm after 10 days. These values were respectively 20.55 ± 1.41% and 27.75 ± 1.93% after 16 days. At both time points, the following trends were seen: T > SWT (*p* < 0.05) at 180 rpm, and T < SWT (*p* < 0.05) at 60 rpm.

#### Population dynamics

**Fig. 6:**
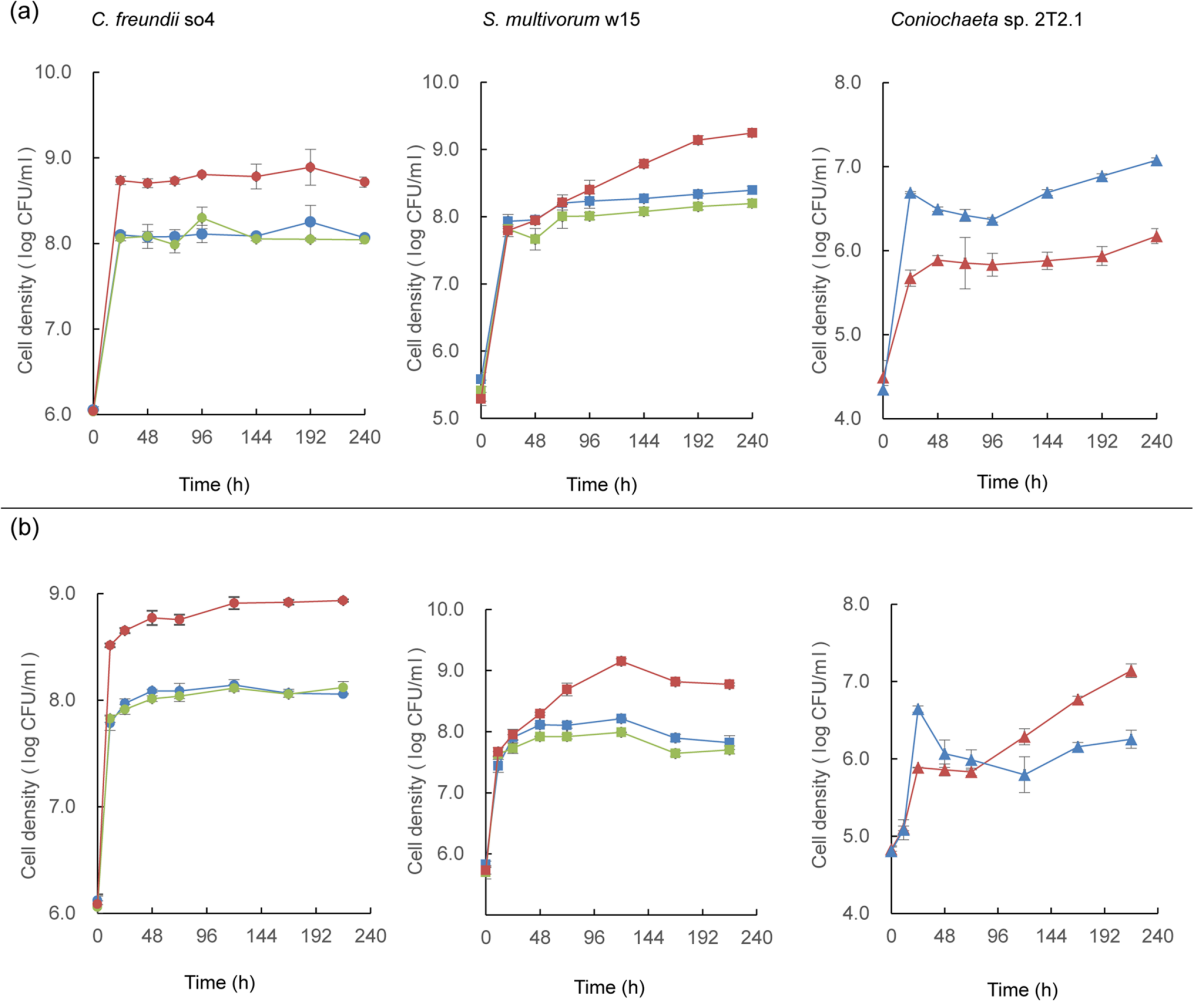
Effect of shaking speed on growth dynamics in synthetic consortia. (a) 180 rpm; (b) 60 rpm. Left: *Citrobacter freundii* so4 (circle); middle: *Sphingobacterium multivorum* w15 (square); right: *Coniochaeta* sp. 2T2.1 (triangle). Blue: strain growing in monoculture; green: strain growing in biculture of *C. freundii* so4 and *S. multivorum* w15; red: strain growing in consortium of strains so4, w15, and 2T2.1

In the 180-rpm cultures, the growth patterns of all strains were similar to those described in the section “WS degradation and population dynamics in synthetic tri- (SWT), bi- (SW), and monoculture (S, W, T) degrader cultures — effect of pH”/ “Population dynamics in the different treatments”/“pH 6.2”. Thus, under the established conditions, both bacteria developed enhanced cell numbers in the presence of the fungus, whereas fungal growth was suppressed (Fig. 6a). Conversely, differential effects were found across the 60-rpm cultures (Fig. 6b). In the respective SWT cultures, strain so4 showed an initial (0–24 h) μ of 0.1040 ± 0.0046, followed by a lower one (0.0014 ± 0.0002) in the following period; the so4 CFU densities stabilized at about 1 × 10^9^ CFU/mL. Strain w15 had an initial (0–24 h) μ of 0.0906 ± 0.0020, versus 0.0039 ± 0.0004 after 24 h. The growth rate of strain so4 in SWT exceeded that in the corresponding SW cultures (*p* < 0.05) after 24 h, but remained similar at later stages; strain w15 in SWT showed initial (0–24 h) growth rates similar to those in the SW cultures, but then showed significantly (*p* < 0.05) higher growth rates at later stage. In the SWT cultures, strain 2T2.1 showed an initial (0–24 h) μ of 0.0445 ± 0.0008 (increasing from about 8 × 10^4^ to about 9 × 10^5^ CFU/mL) (Fig. 6b). This contrasted with its initial μ in culture T, of 0.0778 ± 0.0030 (resulting in population size increases from about 8 × 10^4^ to about 6 × 10^6^ CFU/mL).

Thus, at 60 rpm, both strains so4 and w15 were significantly (*p* < 0.05) stimulated by strain 2T2.1 in the SWT cultures (as compared to culture SW), whereas strain 2T2.1 was initially slightly depressed (∼9 × 10^5^ CFU/mL) as compared to the corresponding monoculture T (∼6 × 10^6^ CFU/mL). This trend reversed after 24 h, as strain 2T2.1 showed a μ of 0.0072 ± 0.0006 in the SWT, against − 0.0009 ± 0.0002 in the T cultures. The strain 2T2.1 cell numbers in culture T went to ∼1 × 10^6^ CFU/mL after 72 h, and became stable at ∼2 × 10^6^ CFU/mL, whereas they increased to ∼1 × 10^7^ CFU/mL in the SWT consortium (after 10 days; Fig. 6b).

#### Different population size increases between 60 and 180 rpm

Given the aforementioned reduced strain 2T2.1 cell densities in the later stages of the 60-rpm T cultures (Fig. 6b) versus the higher ones in the SWT ones, we surmised strains so4 and w15 had fungal-growth-modulating roles under these conditions (Fig. 6b), indicating a switch of roles in the bacterial–fungal consortium.

## Discussion

In this study, we first examined to which extent a novel three-partner bacterial–fungal consortium (SWT), containing three genome-sequenced partners (the LCB-degradative fungus *Coniochaeta* sp. 2T2.1; Mondo et al. 2019 and two bacteria, i.e., *C. freundii* so4 and *S. multivorum* w15; Cortes-Tolalpa et al. 2020), can mimic the WS degradation behavior of a complex soil-derived WS degrading consortium. In a subsequent phase, the effects of selected culture conditions (temperature, pH, and shaking speed) on the growth and the WS degradative performance of the synthetic consortium members were studied using liquid shaken cultures.

### A constructed bacterial–fungal consortium matches the WS degradation activity of a complex soil-derived consortium, and *Coniochaeta* sp. 2T2.1 has a major effect

Clearly, the degradation efficiency, as well as the population build-up of the SWT consortium, matched or surpassed those exhibited by the complex forest soil-derived microbial consortium T10. This was initially found in cultures at pH 7.2, but the effect extended to those run at pH 6.2 and 5.2 (Fig. 1 and Fig. 2). In the process, pH was clearly determinative, with a lowering of pH spurring WS degradation. In effect, the WS degradation efficiencies of the SWT consortia at pH 5.2 and pH 6.2 amounted to over 22.4% of the original weight (Fig. 2), with the degradation efficiencies of the SW consortia being significantly lower. A key underlying reason for this efficiency enhancement was the growth and activity of strain 2T2.1 (22.4% weight loss in consortia with strain 2T2.1 compared to 8.2% in those without strain 2T2.1, Fig. 2 and Fig. 4), lending support to the notion of a key role of this fungus in the process. In previous reports, other fungi (*Fusarium solani*, *Penicillium chrysogenum*) showed similar WS degradation activities, as reflected in the reported weight losses, albeit only after 28 days (Rodriguez et al. 1996). Here, our strain 2T2.1-containing synthetic consortium reached such degradation efficiencies in less than half the time. Whereas the degradation efficiency was pH dependent for the triculture SWT, no such pH dependency was found for the T10 consortium; this indicated a narrow pH range for SWT versus resilience towards pH variation for T10. These findings are consistent with work by Liang et al. (2017), who showed microbial consortium OEM1 (consisting of 31 strains) had largely similar rice straw degradation efficiencies at pH values ranging from 5.0 to 8.0. Similar results were obtained with a microbial consortium used for the treatment of polluted water (Obahiagbon et al. 2014).

Here, the likely major role of *Coniochaeta* sp. 2T2.1 in LCB degradation processes was also supported by the consistent selection of C*oniochaeta* spp. in microbial consortia grown on LCB (Jiménez et al. 2014; Cortes-Tolalpa et al. 2016). Moreover, C*oniochaeta* spp. were previously also found to be key organisms in the detoxification and (partial) degradation of torrified grass, with concomitant stimulation of bacterial growth (Trifonova et al. 2009). A suite of LCB degradation studies performed in our, as well as other, laboratories shows C*oniochaeta* spp. to be able to serve as “deliverers” of dedicated cellulases, xylanases, and lignin peroxidases on different LCB substrates (Ravindran et al. 2012; Lopez et al. 2007; Mondo et al. 2019). Hence, it is likely that, in our newly constructed microbial systems, strain 2T2.1 delivers the enzymes required for key steps in the degradation of WS moieties, such as galactose oxidase, laccase-like enzymes, and multicopper oxidases. Moreover, a dependency of this enzyme delivery on prevailing pH is suggested, although this aspect is as yet unclear.

### *C. freundii* so4 and *S. multivorum* w15 have “accessory” roles in WS degradation, depending on conditions

Given the major role of *Coniochaeta* sp. 2T2.1 in the WS degradation process, one may question what the role of *C. freundii* so4 and *S. multivorum* w15 in the SWT consortium might be. Why do such bacteria consistently show high abundances in the experiments, as well as in the original soil-derived degrader consortia, i.e., T10 (Cortes-Tolalpa et al. 2016)? To shed light on these questions, we examined the population dynamics and potential role of each strain within the different cultures, with a focus on the pH-6.2 cultures at high shaking speed.

#### Division of labor in triplicate consortia: niches and niche occupancy

Given that, in the presence of strain 2T2.1, both *C. freundii* so4 and *S. multivorum* w15 showed rapid initial growth on the WS (achieving much higher population densities than the fungus in each treatment), we posit that the WS-degradative systems offer initial niches suitable for the development of, next to strain 2T2.1, both bacteria. The subsequent slow growth phases were consistent with a postulated niche shift for both bacteria. Basically all systems revealed this dichotomic behavior, and hence the postulated temporal separation of “niches” that were occupied. The niche concept encompasses aspects of both function and condition, and hence we explore these for the systems under study. In other words, how might both nutritional and conditional aspects in the cultures, and shifts therein, have driven the bacterial constituents? Besides initial bacterial growth at the expense of easily degradable compounds from the WS substrate, further nutrients will have increasingly come from activity of *Coniochaeta* sp. 2T2.1, and potentially from either bacterium. Strain 2T2.1 may have primarily attacked substrates like cellulose, next to other bonds between molecules, via secretion of enzymes such as cellulases, laccase-like enzymes, and multicopper oxidases. Indeed, degradation of arabinoxylan, xyloglucan, and cellulose has been identified as key metabolic processes in strain 2T2.1 growing on WS. However, we recently found strain 2T2.1 to overexpress multicopper oxidases and a laccase-like enzyme in the presence of the bacteria (Jiménez et al. 2020), while simultaneously downregulating its (hemi)cellulases. Hence, a role in lignin degradation (15–20% of the WS substrate) is likely. Conversely, the two bacteria might, next to directly attacking the substrate, mainly live off transformation products of the former processes, potentially also relieving feedback inhibitions of fungal functions. With respect to direct attacks on the WS polymers, a suite of genetic systems encoding relevant enzymes involved in hemicellulose degradation was found in the *S. multivorum* w15 genome, whereas the enzyme palette was very restricted in *C. freundii* so4 (Cortes-Tolalpa et al. 2020). Thus, strain so4, being a facultatively anaerobic bacterium with a wide pH tolerance range (4–10; Supplemental Fig. S1a), has the capacity to grow on carbohydrates such as diverse amino acids, carboxylic acids, and sugar alcohols (as evidenced by analyses of its genome as well as functional tests). It also contains genes for consumption of putrescine, and so may have had a role in detoxification of the systems (Cortes-Tolalpa et al. 2020). On the other hand, *S. multivorum* w15, being strictly aerobic and non-fermentative, can grow at pH 5–9 (Supplemental Fig. S1b), resulting in the inviability of strain w15 in SWT consortium at initial pH 5.2 (Fig. 4c). The pH decreases may have been caused by acid metabolites produced by any of the organisms, including the fungus; for instance, xylo-oligomers from the WS may have been decomposed to acetic acid/acetate (Shahab et al. 2020). On the basis of existing data, strain w15 is a very versatile organism, being able to thrive on mono-sugars (e.g., glucose, arabinose, cellobiose, glycerol, fructose, and xylose; Holmes et al. 1981); di-, tri-, and tetra-saccharides; starch (dextrin, α-, β- and γ-cyclodextrins); and diverse polysaccharides such as pectin and inulin (Cortes-Tolalpa et al. 2020). Indeed, the genome of *S. multivorum* w15 revealed a plethora of genes encoding proteins of diverse CAZy families and carbon-binding modules (CBMs), many of which are associated with (hemi)cellulose degradation (Cortes-Tolalpa et al. 2020). We surmised that *S. multivorum* w15, given its genomic armory, mainly focused on degrading (hemi)cellulose, making simple sugars available in the system. This is consistent with previous results on similar consortia grown on WS (Cortes Tolalpa et al. 2017).

Moreover, as genes encoding (hemi)cellulases in *Coniochaeta* sp. 2T2.1 may have been repressed in the presence of the two bacteria (Jiménez et al. 2020), a key niche (involving specific (hemi)cellulose bond breaks) may be occupied by bacterial function. This is consistent with the finding that enzymes of relevant CAZy classes can be produced, e.g., GH5 by strain so4; GH9, GH29, and GH92 by strain w15; and GH2 and GH43 by both bacteria (Cortes-Tolalpa et al. 2020).

#### How do culture conditions shape the dynamics of the three SWT consortium partners?

The clear effects of pH as well as shaking speed on both WS degradation and population dynamics in the SWT consortia pointed at key niche shifts across the three consortium members. Whereas the effects of pH were consistent with our understanding of their pH tolerance ranges, as discussed in the foregoing, those of shaking speed are potentially more intricate, as they encompass aspects of heterogeneity and oxygen diffusion. Briefly, one can posit that the differential heterogeneity observed between the 180-rpm and the 60-rpm cultures is at the basis of the population dynamics and degradation value effects, as it includes aspects of both shifted spatial distributions of compounds and cells, and of local oxygen levels and dynamics. Compared to the 60-rpm shaking speed, the fast shaking may have jeopardized cellular adherence to, and settlement on, the WS particles, resulting in modified biofilm formation. Moreover, there may have been differential local compound and oxygen diffusion rates and levels. Hereunder, we examine the data regarding such (combined) effects.

### Shaking speed affects a niche shift between *C. freundii* so4, *S. multivorum* w15, and *Coniochaeta* sp. 2T2.1

Our observation of the strong effect of shaking speed — resulting in overall shifted oxygen levels — in the fungal monoculture T was consistent with data from the literature, in which higher dissolved oxygen levels in microbial cultures will generally result in higher secreted enzyme levels. For instance, Tuncer et al. (1999) reported such effects on extracellular endoxylanase, endoglucanase, and peroxidase levels in *Thermomonospora fusca* BD25. Also, Lopez et al. (2007) showed that enzymes produced by *C. ligniaria* NRRL 30,616 after 5 days were at higher levels in cultures shaken at 120 rpm than in semisolid-state ones. Thus, conditions that affect, next to heterogeneity, the levels of oxygen are key drivers of fungal LCB degradation activities. Moreover, several studies have examined the roles of bacteria, in combination with fungi, in the degradation of lignocellulose (Trifonova et al. 2009; Shahab et al. 2020). Recent studies also identified oxygen level as an important bacterial–fungal interaction modulator, particularly for *Candida albicans*–bacteria interactions (Deveau et al. 2018). Suwannarangsee et al. (2012) combined the (hemi)cellulolytic activities in *Aspergillus aculeatus* BCC199 with *Bacillus subtilis* expansin (non-catalytic protein that can increase the hydrolysis of lignocelluloses by loosening the plant cell wall) to enhance rice straw degradation (Suwannarangsee et al. 2012). Spatially heterogeneous conditions, with strong oxygen gradients, were also used by Shahab et al. (2020) in a membrane reactor. They found fair LCB degradation to short-chain fatty acids by the cellulolytic fungus *Trichoderma reesii* (growing in an oxygen-rich spatial niche), making glucose and xylose available to the system, a (facultatively anaerobic) lactic acid bacterium funneling these to lactate, and a lactate-consuming anaerobic bacterium for product formation (Shahab et al. 2020).

How about the conditions in our bacterial–fungal (SWT) versus the T cultures? Can the differential oxygen concentrations in the SWT versus the T cultures (shaking speeds 180 versus 60 rpm) per se explain the different growth dynamics? Here, the finding of bacterial suppression of fungal growth in SWT at 180 rpm (comparison SWT to T) versus enhanced fungal growth in such cultures at 60 rpm was striking (Fig. 6). Clearly, at 60 rpm, the physical appearance of “T” cultures was different (containing particle agglomerates yet appearing more transparent) from that in the SWT ones (being cloudy), whereas this was not found at 180 rpm. Given that *Coniochaeta* spp. in liquids can show “strongly adherent” phenotypes (potentially through a secreted compound coined the “glue”; Epstein and Nicholson, 2006), we hypothesized that the presence of the bacterial strains affected the production or secretion of such a compound, or interrupted the adhesion. These so-called fungal glues may consist of glycans or derivatives; e.g., β-glucans are an essential component of the matrix in *C. albicans* biofilms (Lipke 2018). Since the two bacteria are capable of producing various enzymes attacking glycans (Cortes-Tolalpa et al. 2020), the adhesive/agglomerative properties of *Coniochaeta* may have been affected in the SWT cultures by enzyme-driven mechanisms. Thus, in the 60-rpm SWT cultures, bacterial activities (in particular those of so4) may have modulated fungal biofilm formation, resulting in higher oxygen availability around the fungal biomass. This is in contrast to the T cultures, in which the biofilms may have limited oxygen supply, resulting in slowed growth and WS degradation activity.

In all cultures, heterogeneity has probably established gradients of not only oxygen but of a range of (intermediate and product) compounds. Such heterogeneity is an important factor driving all interactions within our consortia. Spatiotemporally heterogeneous conditions affecting fungal–bacterial interactions at different points in time during development have been discussed by Deveau et al. (2018). Given that the data on population dynamics obtained by us are “overall,” the underlying effects of heterogeneity are not easily visible. For instance, the overall growth of strain 2T2.1 was apparently suppressed by the bacteria at high oxygen levels, whereas it was “rescued” by the same bacteria under limited oxygen levels. It is logical to suppose that *C. freundii* so4, as a facultative anaerobe, had a key role in providing metabolic “helper” functions at 60 rpm (resulting in lowered oxygen levels), by:

1. Removing inhibitory substances under conditions of low oxygen, and/or.
2. Producing intermediate metabolites which could be used by *S. multivorum* w15 and *Coniochaeta* sp. 2T2.1, and/or.
3. Shifting to anaerobic respiration or even fermentation, thus modifying its niche and reducing the competition for oxygen exerted by the other organisms.

This is in line with the hypothesis that — within heterogeneous microbial consortia — conditions in microenvironments surrounding the cell agglomerates are strongly determinative for the system; these can be modulated by one partner to the benefit of another one (which is beneficial to the whole). This way, a facultative anaerobe can have a key function under low oxygen tension, serving another function in aerobic ones. This as a return for the soluble sugars released via cellulose hydrolysis (Zuroff and Curtis, 2012). In future work, transcriptomics analyses should be applied to explain the divergent behavior of consortium members between the high and low shaking speeds.

## Supplementary Information

Below is the link to the electronic supplementary material.

Supplementary file1 (PDF 263 KB)

## Data Availability

The datasets generated during and analyzed during the current study are available from the corresponding author on reasonable request.
