# Supplementary material for: Effect of culture conditions on the performance of lignocellulose-degrading synthetic microbial consortia
Source: Appl Microbiol Biotechnol. 2021 Oct 1;105(20):7981–95. doi: 10.1007/s00253-021-11591-6 (PMC8502130; doi:10.1007/s00253-021-11591-6)
Supplement: Supplementary file 1 — Supplementary file1 (PDF 263 KB) [file 253_2021_11591_MOESM1_ESM.pdf]

Applied Microbiology and Biotechnology

Effect of culture conditions on the performance of lignocellulose-degrading synthetic microbial consortia

Yanfang Wang[1*], Theo Elzenga[2], Jan Dirk van Elsas[1*]

1 Cluster of Microbial Ecology, Groningen Institute for Evolutionary Life Sciences, University of Groningen, Groningen, the Netherlands
2 Plant physiology group, Groningen Institute for Evolutionary Life Sciences, University of Groningen, Groningen, the Netherlands

*** Correspondence:**
Jan Dirk van Elsas
j.d.van.elsas@rug.nl

Yanfang Wang
wang-yanfang@qq.com

pH tolerance range of growth of each strain

The pH tolerance of each strain was determined by growing the bacterial strains in Lennox medium ((Sigma-Aldrich, Darmstadt, Germany)) at different pH (3, 4, 5, 7, 9 10). The optical density (OD) at 600 nm of the bacterial cultures were measured at every 2 hours with a microtiter plate reader set at 600 nm wavelength. The value of optical density at 600 nm (OD600) was used as cell density.

Citrobacter freundii so4 grew at a wide pH range: pH 5.0, 6.0, 7.0, 8.0, 9.0, 10.0, was inhibited at pH 4.0, and didn't survive at pH 3.0 (Supplemental Fig. S1a). *S. multivorum* w15 grew between pH 5.0 and 7.0, optimally at pH 7.0, was highly inhibited at pH 9.0, and didn't survive at pH 3.0, 4.0 and 10.0 (Supplemental Fig. S1b).

Comparison of growth on washed and unwashed wheat straw

In order to reduce the effect of easily removable small molecules on growth, we compared the dynamics of the consortium components by comparing growth on washed and unwashed WS. After 24 hours of cultivation, cell densities in consortia with washed WS increased to ~ 8.5 log cells/mL, compared to that of unwashed WS (~ 9.1 log cells/mL; Supplemental Fig. S2). Thus, many water-soluble compounds, e.g. sugars, L-arabinose and lactose, were probably removed by the WS washing. The rapid increases of the population sizes indicated the WS degradation processes by the strains.

Supplementary figures

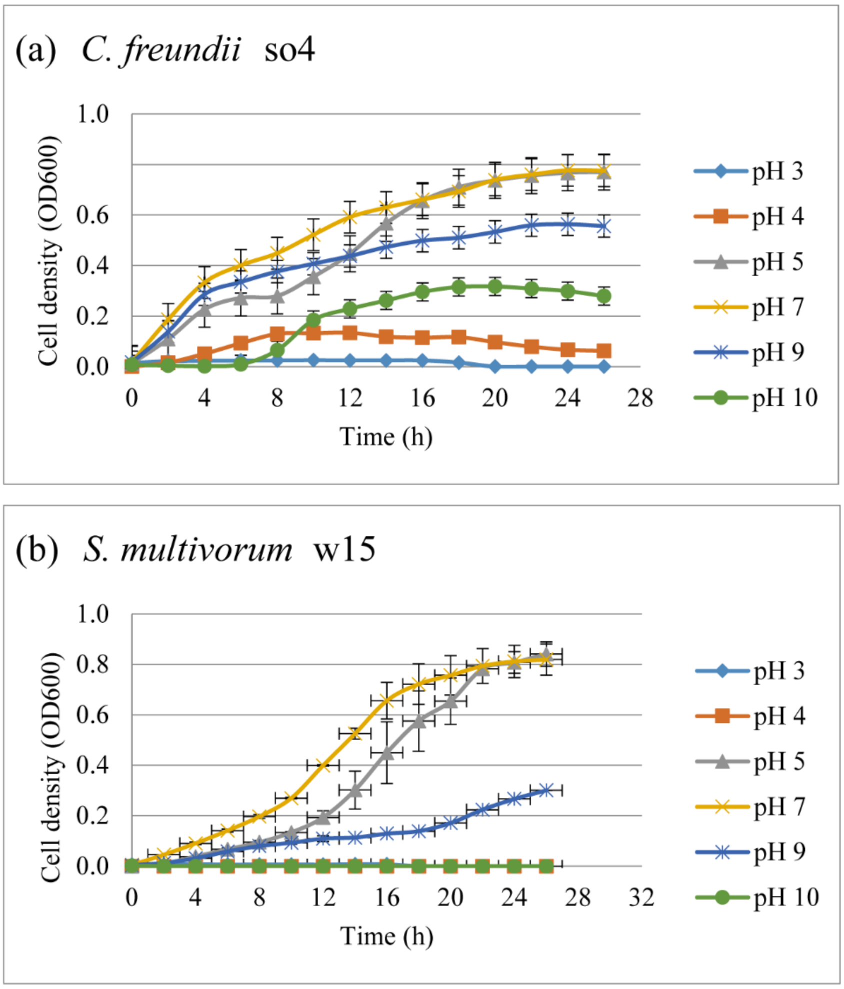

Supplemental Fig. S1 - pH range of growth of (a) *Citrobacter freundii* so4 and (b) *Sphingobacterium multivorum* w15

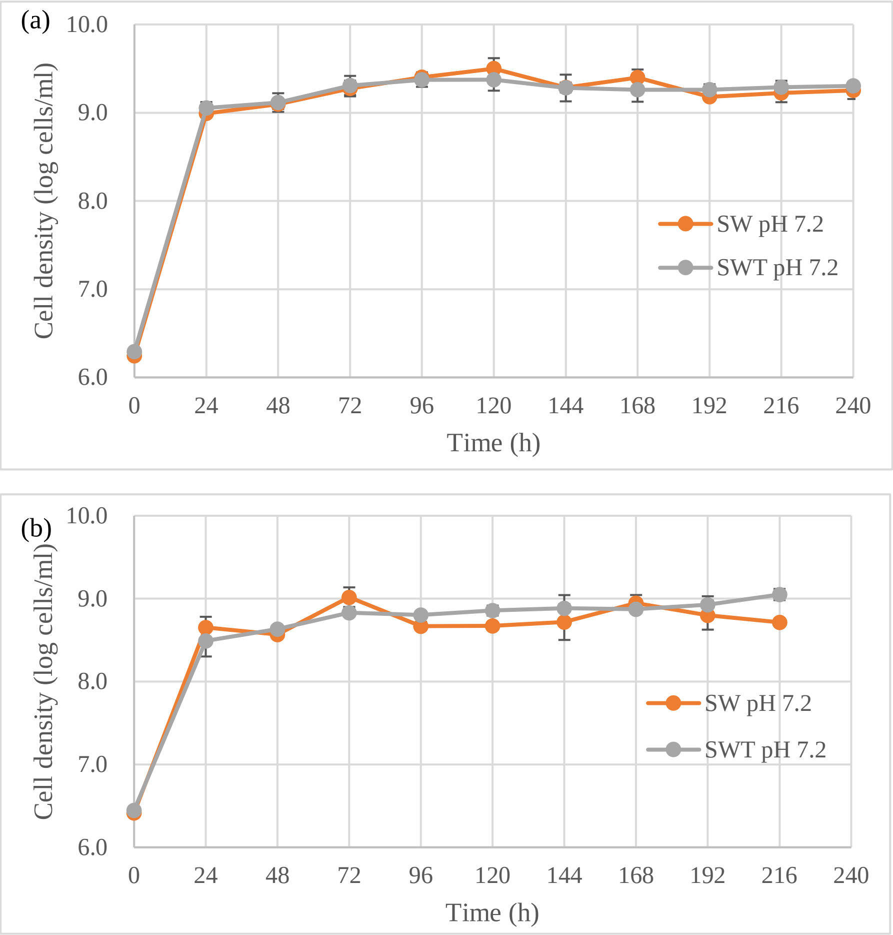

Supplemental Fig. S2 - Dynamics of growth using unwashed (a) and washed (b) wheat straw at pH 7.2
Abbreviations: SW: Consortia of two bacteria (*Citrobacter freundi* so4 + *Sphingobacterium multivorum* w15); SWT: Consortia of two bacteria plus the fungus (so4+w15 + *Coniochaeta* sp. 2T2.1)

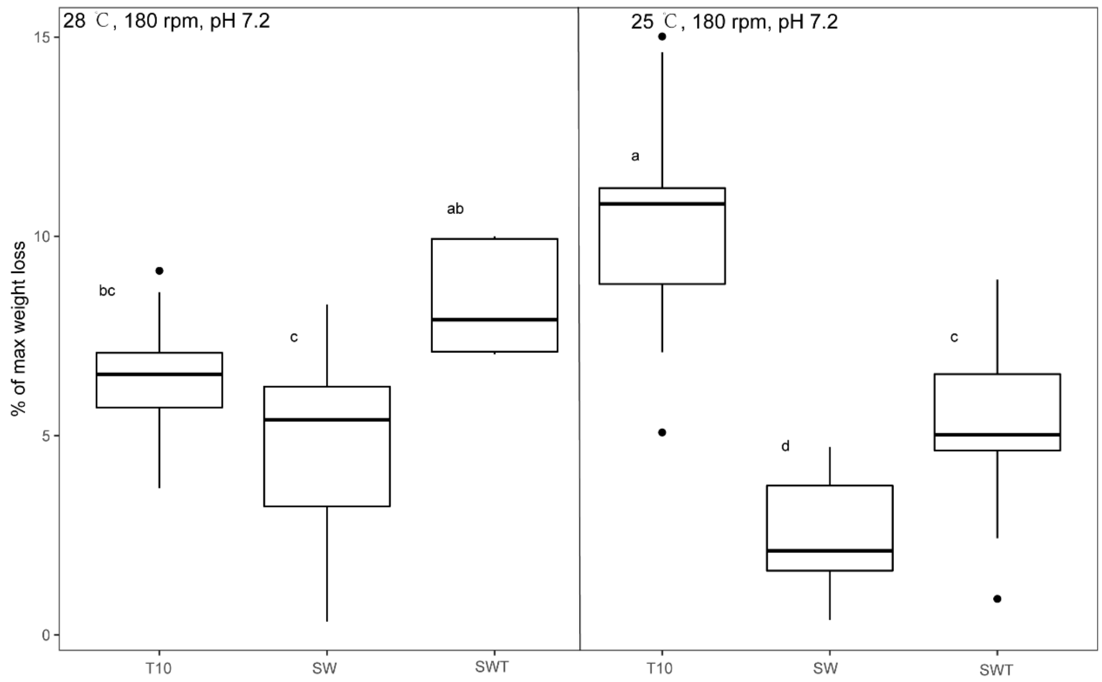

Supplemental Fig. S3 Degradation performance of each consortium at tested temperature (28 °C and 25 °C)

Abbreviations: T10: forest soil-derived LCB-degrader consortium (10 transfers; Cortes-Tolalpa et al. 2016); SW: Consortium consisting of *Citrobacter freundii* so4 and *Sphingobacterium multivorum* w15; SWT: Consortium of strains so4, w15 and *Coniochaeta* sp. 2T2.1

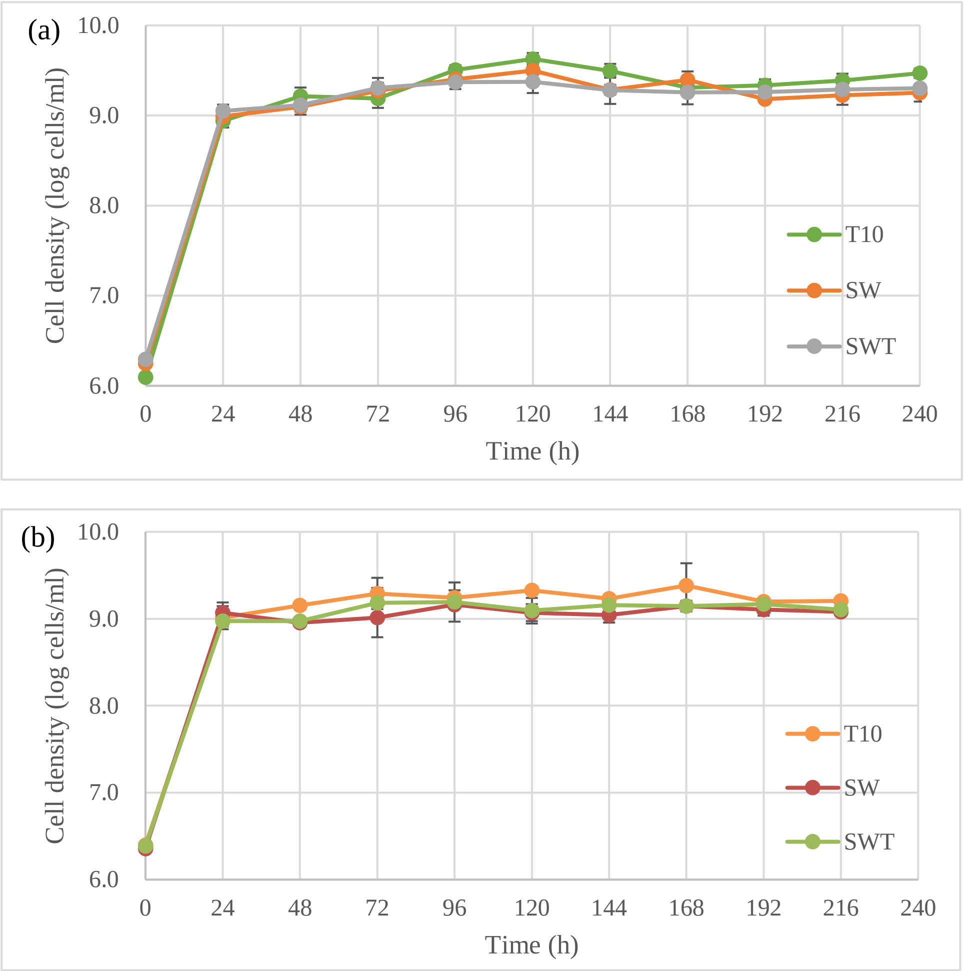

Supplemental Fig. S4 Growth dynamics of cells in each treatment at tested temperature (a) 28 °C, (b) 25 °C at pH 7.2

Total cell number were counted, overall bacterial cells.

Abbreviations: T10: LCB-degrader communities driven from soil (Cortes-Tolalpa et al. 2016); SW: Consortia of two bacteria (*Citrobacter freundi* so4 + *Sphingobacterium multivorum* w15); SWT: Consortia of two bacteria plus the fungus (so4+w15 + *Coniochaeta* sp. 2T2.1)

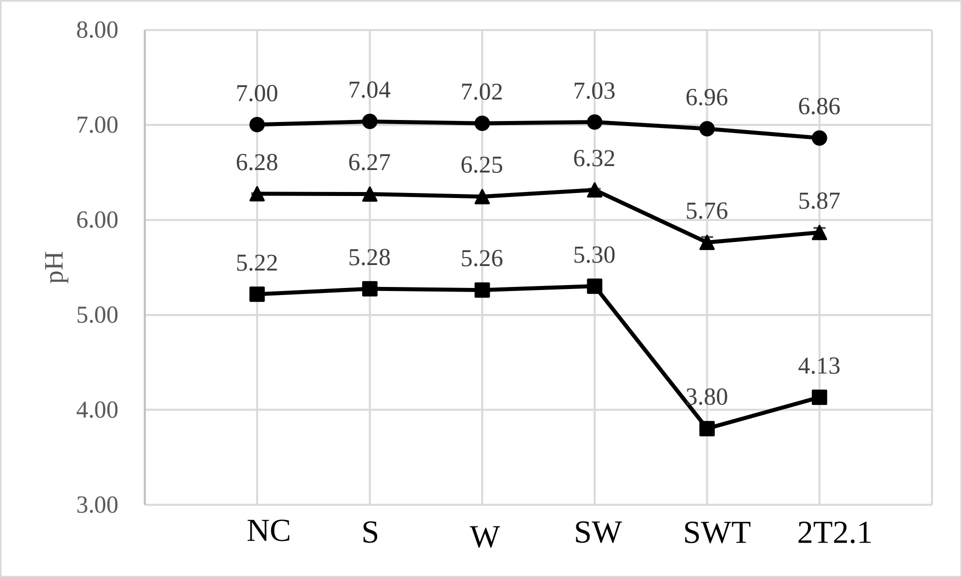

Supplemental Fig. S5 Final pH value (at the end of the culture, day 10) at 180 rpm with an initial pH of 7.2 (circle), 6.2 (triangle) and 5.2 (square)

Abbreviations: NC: Negative control; S: Monoculture of *Citrobacter freundi* so4; W: Monoculture of *Sphingobacterium multivorum* w15; SW: Consortium of two bacteria (so4 + w15); 2T2.1: Monoculture of fungus *Coniochaeta* sp. 2T2.1; SWT: Consortium of two bacteria plus the fungus (so4+w15 + 2T2.1)

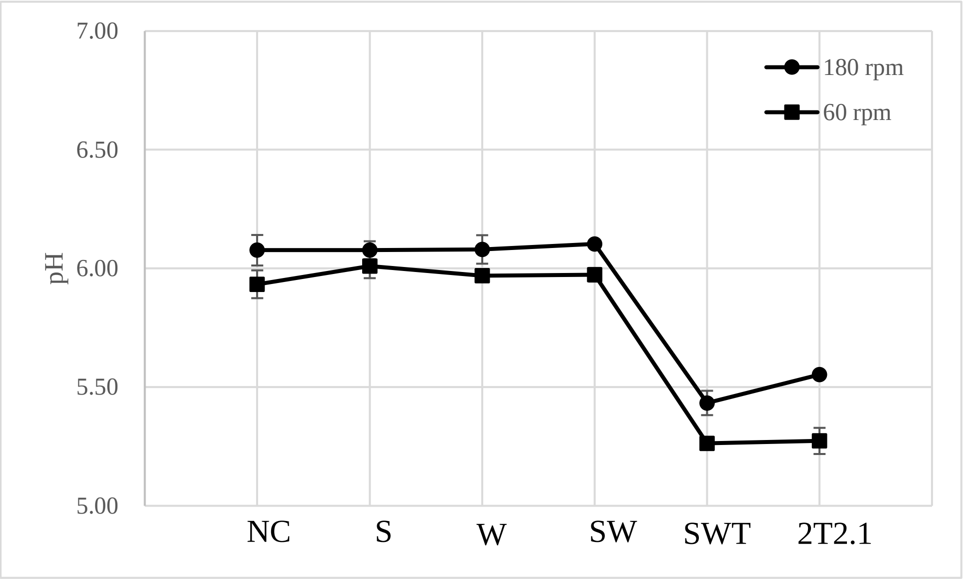

Supplemental Fig. S6 Final pH value at the end of the culture at 60 rpm (square) and 180 rpm (circle) with an initial pH of 6.2

Abbreviations: NC: Negative control; S: Monoculture of *Citrobacter freundi* so4; W: Monoculture of *Sphingobacterium multivorum* w15; SW: Consortium of two bacteria (so4 + w15); 2T2.1: Monoculture of fungus *Coniochaeta* sp. 2T2.1; SWT: Consortium of two bacteria plus the fungus (so4+w15 + 2T2.1)

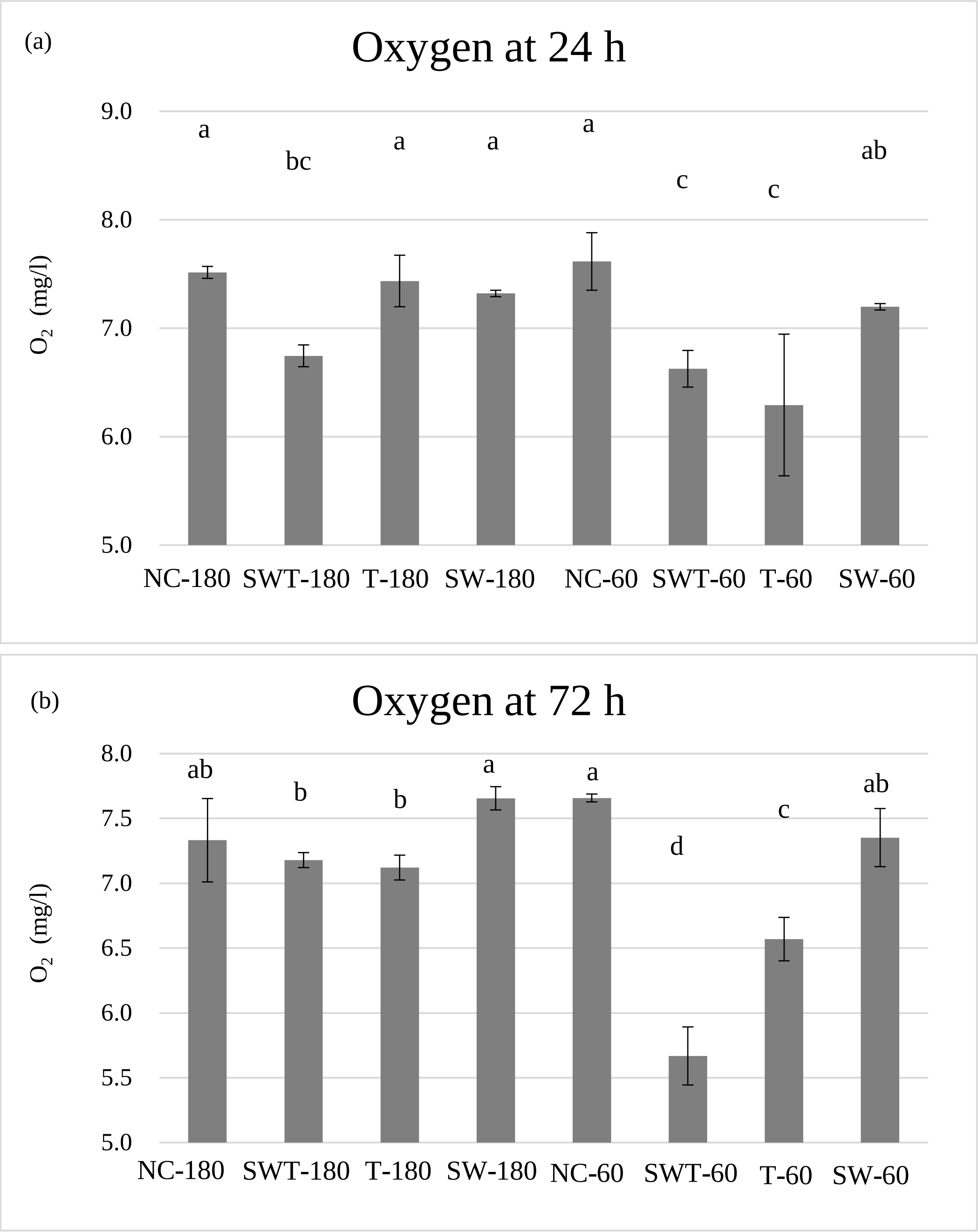

Supplemental Fig. S7 Dissolved oxygen concentrations in different treatments at 180 rpm and 60 rpm at (a) 24 h and (b) 72 h

Abbreviations: NC: Negative control; SW: Consortium of two bacteria (*Citrobacter freundi* so4 + *Sphingobacterium multivorum* w15); T: monoculture of fungus *Coniochaeta* sp. 2T2.1; SWT: Consortium of two bacteria plus the fungus (so4+w15 + 2T2.1); -180: 180 rpm; -60: 60 rpm

Reference

Cortes-Tolalpa L, Jiménez DJ, de Lima Brossi MJ, Salles JF, van Elsas JD (2016) Different inocula produce distinctive microbial consortia with similar lignocellulose degradation capacity. Appl Microbiol Biotechnol 100: 7713-7725
